# Vaccination

**Published:** 1878-03

**Authors:** 


VACCINATION.—Jenner's great discovery of preventing small-pox by vaccination, gives mankind the power of avoiding one of the most painful, loathsome and dangerous diseases to which people generally are subject. Strange to say, however, many absolutely refuse this almost infallible safeguard against the disease, depending on keeping aloof from those affected. This seeming recklessness of health and even life may be, in part, accounted for in the fact that spurious vaccination sometimes leads to very unpleasant consequences without affording the desired protection. It is, therefore, important that physicians exercise great caution in securing reliable vaccine virus.

In order to protect her citizens against small-pox and against the too frequent liability to spurious vaccination, the State has made provision for a supply of genuine virus. To this end the Governor has directed that physicians throughout the State be supplied free of charge by addressing J. G. WESTMORELAND, M.D.,
14 Loyd street, Atlanta, Ga.

